# Intertwined Signaling Pathways Governing Tooth Development: A Give-and-Take Between Canonical Wnt and Shh

**DOI:** 10.3389/fcell.2021.758203

**Published:** 2021-10-29

**Authors:** Florian Hermans, Lara Hemeryck, Ivo Lambrichts, Annelies Bronckaers, Hugo Vankelecom

**Affiliations:** ^1^Laboratory of Tissue Plasticity in Health and Disease, Cluster of Stem Cell and Developmental Biology, Department of Development and Regeneration, Leuven Stem Cell Institute, KU Leuven (University of Leuven), Leuven, Belgium; ^2^Biomedical Research Institute (BIOMED), Department of Cardio and Organ Systems, UHasselt-Hasselt University, Diepenbeek, Belgium

**Keywords:** tooth, Wnt, β-catenin, sonic hedgehog (Shh), odontogenesis, stem cells

## Abstract

Teeth play essential roles in life. Their development relies on reciprocal interactions between the ectoderm-derived dental epithelium and the underlying neural crest-originated mesenchyme. This odontogenic process serves as a prototype model for the development of ectodermal appendages. In the mouse, developing teeth go through distinct morphological phases that are tightly controlled by epithelial signaling centers. Crucial molecular regulators of odontogenesis include the evolutionarily conserved Wnt, BMP, FGF and sonic hedgehog (Shh) pathways. These signaling modules do not act on their own, but are closely intertwined during tooth development, thereby outlining the path to be taken by specific cell populations including the resident dental stem cells. Recently, pivotal Wnt-Shh interaction and feedback loops have been uncovered during odontogenesis, showing conservation in other developing ectodermal appendages. This review provides an integrated overview of the interplay between canonical Wnt and Shh throughout mouse tooth formation stages, extending from the initiation of dental placode to the fully formed adult tooth.

## Introduction

### Mouse Tooth Development

**FIGURE 1:**
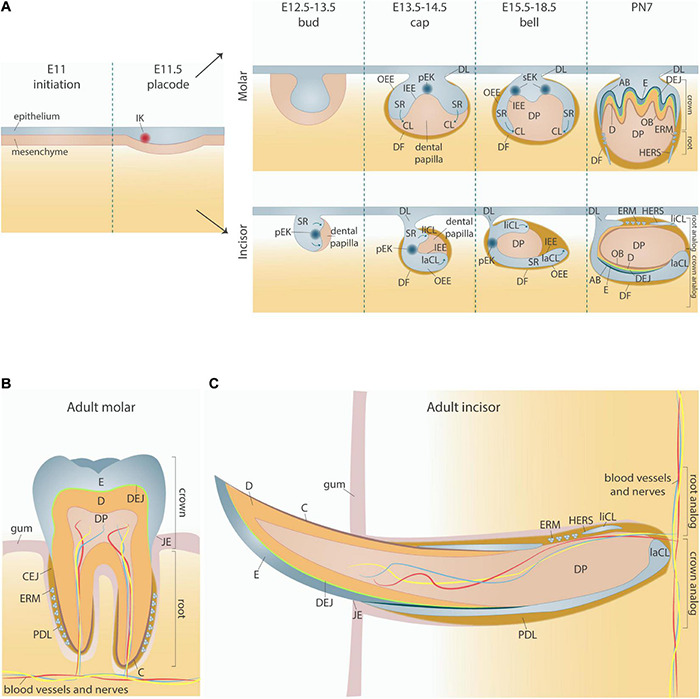
Overview of mouse tooth development. **(A)** Embryonic stages of mouse tooth development, with divergent morphology of molar and incisor tooth germs. **(B,C)** Schematic of fully formed adult molar **(B)** and incisor **(C)**. AB, ameloblasts; C, cementum; CEJ, cemento-enamel junction; CL, cervical loop; D, dentin; DEJ, dentino-enamel junction; DF, dental follicle; DL, dental lamina; DP, dental pulp; E, enamel; E11, embryonic day 11; ERM, epithelial cells rests of Malassez; HERS, Hertwig’s epithelial root sheath; IEE, inner enamel epithelium; IK, initiation knot; JE, junctional epithelium; laCL, labial cervical loop; liCL, lingual cervical loop; pEK, primary enamel knot; PDL, periodontal ligament; PN7, postnatal day 7; OB, odontoblasts; OEE, outer enamel epithelium; SR, stellate reticulum.

Mouse tooth development initiates around embryonic day 11 (E11), when localized epithelial thickenings in the oral ectoderm form and establish the molar and incisor dental placodes at E11.5 (Figure 1). Subsequently, the dental epithelium proliferates and invaginates into the underlying mesenchyme which condenses around the epithelium to form the tooth bud (E12.5–E13.5). Over the following days, the epithelium continues to extend around the dental mesenchyme, thereby forming a cap (visible at E13.5–E14.5) and later a bell shape (E15.5–E18.5). During the bud-to-cap transition, the developing molar and incisor tooth germs essentially require signals from the primary enamel knot (pEK), a transient signaling center located in the dental epithelium (Jernvall et al., 1994). This pEK, morphologically distinct with densely packed non-dividing (G1-phase) cells, is characterized by expression of the cyclin-dependent kinase inhibitor p21 (*Cdkn1a*) and secretion of a variety of signaling molecules, in particular members of the sonic hedgehog (Shh), wingless-type MMTV integration site (Wnt), fibroblast growth factor (FGF), and bone morphogenetic protein (BMP) families, that affect the surrounding epithelium and mesenchyme (Ahrens, 1913; Jernvall et al., 1994, 1998; Vaahtokari et al., 1996; Thesleff and Sharpe, 1997).

Although the pEK was the first signaling hub to be discovered during odontogenesis, an earlier signaling center is already established upon invagination of the dental placode (E11.5), termed the initiation knot (IK). First described in incisors (Ahtiainen et al., 2016) and only recently in molars (Mogollón et al., 2021), the IK consists of non-mitotic (G1-phase) *Cdkn1a*-expressing cells, thus comparable to the pEK. Moreover, identical signaling molecules were found in pEK and IK (Ahtiainen et al., 2016; Mogollón et al., 2021). Whereas the pEK is crucial for the bud-to-cap transition, the IK is thought to play a key role in the preceding placode-to-bud progression. Mice lacking *Eda* (*Tabby* mice), which encodes a transmembrane protein of the tumor necrosis factor (TNF) family and an important regulator of tooth size and shape (Grüneberg, 1965; Pispa et al., 1999), show a reduced IK size leading to smaller tooth buds (Ahtiainen et al., 2016). Interestingly, IK cells do not generate or contribute to the pEK which instead forms *de novo* (Ahtiainen et al., 2016; Mogollón et al., 2021).

In response to signals from the pEK, the dental epithelial tissue elongates transversely (molars) or longitudinally (incisors), thus extending into and around the underlying mesenchyme and forming the cervical loops (CLs) on both sides of the condensed mesenchyme, now referred to as the dental papilla (Figure 1A). Whereas molar CLs grow symmetrically around the papilla, incisor CLs extend unevenly along the labial-lingual axis, forming a smaller slow-growing lingual CL (liCL) and a larger labial CL (laCL) which continues to grow throughout incisor development as well as adult life (Yu and Klein, 2020).

At the end of the cap stage, the pEK of both incisors and molars undergoes apoptosis (Jernvall et al., 1998). In monocuspid teeth (such as incisors) the pEK is not replaced, whereas in multicuspid teeth (such as molars) the pEK is substituted by secondary EKs (sEKs) at the bell stage (E15.5) which determine the position and number of the tooth cusps (Figure 1A) (Jernvall et al., 1998; Jernvall and Thesleff, 2012). Recently, it has been demonstrated that some molar pEK cells escape apoptosis and contribute to sEK formation, an idea long disputed previously (Matalova et al., 2005; Ahtiainen et al., 2016; Du et al., 2017; Mogollón et al., 2021). Together, the successive stages in tooth development appear strongly guided by succeeding signaling centers.

During the transition from cap to bell stage, the epithelium folds, and is further compartmentalized into morphologically and functionally distinct inner and outer enamel epithelia (IEE and OEE, respectively). The IEE lies adjacent to the dental papilla, while the OEE is located in the periphery of the future enamel organ (Figure 1A). The zone of epithelial cells sandwiched between the IEE and OEE composes the stellate reticulum (SR), which is uniquely vascularized (Shadad et al., 2019).

Following further invagination and elongation of the CLs into the dental mesenchyme, the crown and root are established, and the dental epithelial and mesenchymal cells further differentiate. These differentiation processes are dependent on intimate epithelial-mesenchymal interplay. Under influence of the IEE, the mesenchyme differentiates into odontoblasts that produce dentin, while the mesenchyme directs the IEE into differentiation toward ameloblasts that produce enamel (Figures 1A–C). The OEE forms the junctional epithelium bridging the tooth surface and oral mucosa (Figures 1B,C).

In molars, the CLs elongate apically into the underlying mesenchyme to form Hertwig’s epithelial root sheath (HERS), a transient bilayer structure involved in epithelial-mesenchymal interactions necessary for tooth root formation and sandwiched in between the dental pulp and follicle (Figure 1A) (Li et al., 2015, 2017). In the mouse incisor, as different from the molar, the laCL contains epithelial stem cells which persist and drive continuous tooth growth throughout life, and the root analog and HERS are restricted to the liCL side (Yu and Klein, 2020). Eventually, HERS disintegrates, and the remaining epithelial cells become dispersed throughout the dental follicle, a thin cell layer ensheathing the developing root, thereby establishing the epithelial cell rests of Malassez (ERM) (Figures 1A–C). In addition to the ERM, the dental follicle also contains mesenchymal cells and extracellular matrix and forms the interface between the tooth root and adjacent bone. Collectively, the dental follicle and HERS/ERM contribute to tooth root formation, tooth eruption, cementum production, periodontal ligament (PDL) formation and anchorage of the tooth in the alveolar bone.

Tooth development is tightly controlled by multiple evolutionarily conserved signaling pathways (Thesleff and Sharpe, 1997). In particular, canonical Wnt and Shh signaling appear crucial for odontogenesis, since disruption in each pathway leads to either impaired tooth formation or supernumerary teeth. Below, after a brief introduction of both pathways, we review in detail their involvement throughout mouse tooth development.

### Canonical Wnt/β-Catenin Signaling

The Wnt pathway can act through canonical β-catenin-dependent signaling and non-canonical signaling (which is not further discussed here) (van Amerongen and Berns, 2006; Tamura and Nemoto, 2016). The mammalian pathway counts 19 Wnt proteins and 10 receptors (Frizzled, Fzd). In the absence of Wnt ligand, β-catenin is complexed in the cytoplasm with axis inhibition protein (AXIN), adenomatous polyposis coli (APC) tumor suppressor, casein kinase 1 (CK1) and glycogen synthase kinase 3β (GSK3β) which phosphorylates β-catenin, thereby targeting the protein for proteasomal degradation (Behrens et al., 1998). Upon binding of canonical Wnt ligands to their cognate Fzd receptors, which are bound to co-receptors of the low-density lipoprotein receptor-related protein family (Lrp4-6), dishevelled (DVL) and AXIN are recruited to the membrane. This recruitment disrupts the β-catenin-degradation complex and allows β-catenin to accumulate and translocate to the nucleus where it complexes with transcription factors of the T-cell-specific factor/lymphoid enhancer binding factor (TCF/LEF) family to regulate expression of target genes. Wnt/β-catenin signaling activity is tightly controlled by numerous factors including specific inhibition by, among others, Dickkopf (Dkk1-4) and secreted Fzd-related protein (Sfrp1-5) family members, sclerostin (Sost), Wnt inhibitory factor 1 (Wif1), sclerostin domain containing 1 (Sostdc1, also known as Wise, ectodin or USAG-1) and Notum. Many of these secreted Wnt antagonists function *via* binding of the Lrp co-receptors (Tamura and Nemoto, 2016). On the other hand, Wnt/β-catenin signaling is activated by R-spondin (Rspo1-4) glycoproteins through binding to their leucine rich repeat containing G protein-coupled receptors (Lgr4-6) which blocks Fzd internalization, thus boosting Wnt’s signaling activity (Carmon et al., 2011; de Lau et al., 2011; Glinka et al., 2011).

### Shh Pathway

Shh is one of the three secreted peptides [in addition to Indian and Desert hedgehog (Hh)] that mediate Hh signaling. In the absence of ligand, the Hh receptor Patched (Ptch) accumulates in the cell membrane and represses the other receptor Smoothened (Smo). Upon binding of Hh ligands to Ptch, the complex is internalized and degraded, thereby terminating Smo inhibition and allowing it to accumulate at the ciliary membrane. This activated Smo leads to the processing and subsequent activation of glioma-associated oncogene (Gli1-3) transcription factors which regulate the downstream expression of Shh target genes. Shh signaling is self-regulating with pathway activity modulating the expression of Ptch and Gli transcription factors as well as of negative regulators such as Hh-interacting protein (Hhip1) and growth-arrest specific 1 (Gas1). Importantly, Sostdc1 is a Shh target gene and at the same time a Wnt inhibitor, supporting a negative feedback loop between Shh and Wnt signaling (Ahn et al., 2010).

## Wnt and Shh Signaling: Crucial Intertwined Regulators of Tooth Development

### Wnt and Shh Specify the Tooth-Forming Fields in Embryonic Oral Ectoderm

**FIGURE 2:**
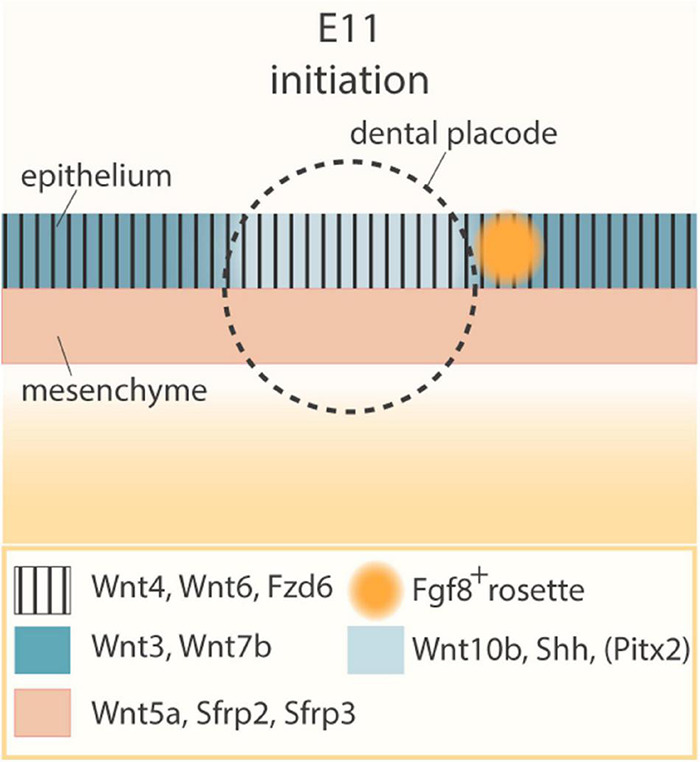
Wnt and Shh pathway expression pattern prior to dental placode formation. Schematic of the E11 initiation stage of tooth development, before formation of the dental placode, and expression pattern of key regulatory genes. The circle indicates the prospective position of the dental placode, established at E11.5. During tooth initiation at E11, *Wnt4*, *Wnt6*, and *Fzd6* (vertical shading) are expressed throughout the oral ectoderm, while *Wnt5a*, *Sfrp2*, and *Sfrp3* are produced in the underlying mesenchyme (peach). In the oral ectoderm, *Wnt3* and *Wnt7b* (dark blue) are expressed in a pattern mutually exclusive with *Wnt10b*, *Shh*, and *Pitx2* (light blue), the latter being restricted to the prospective dental placode. Also, a rosette of oral epithelial cells (orange), characterized by *Fgf8* expression, is located posteriorly to the placode.

Already in the late 90s, it was shown that multiple Wnt pathway genes are expressed during development of the dental placodes (Dassule and McMahon, 1998; Sarkar and Sharpe, 1999). *Wnt10b* is essentially restricted to the dental epithelial thickenings in the oral ectoderm, while *Wnt4*, *Wnt6* and *Fzd6* are expressed throughout the oral ectoderm (Figure 2) (Dassule and McMahon, 1998; Sarkar and Sharpe, 1999). Interestingly, *Wnt3* and *Wnt7b*, both canonical Wnt pathway activators, are also expressed in the oral epithelium but excluded from the prospective dental placodes (Sarkar and Sharpe, 1999). Simultaneously, *Wnt5a* and Wnt antagonists *Sfrp2* and *Sfrp3* are expressed in the underlying mesenchyme (Sarkar and Sharpe, 1999). In addition, Rspo/Lgr signaling members are dynamically expressed throughout the earliest (as well as later) stages of odontogenesis, although their precise roles have not yet been documented (Kawasaki et al., 2014). Wnt/β-catenin pathway activity has indeed been detected using Wnt reporter mouse lines (such as *Tcf/Lef-LacZ*, *TOPGAL*, *BAT-gal* or *Axin2-LacZ*) in the developing dental placodes and the underlying dental mesenchyme, as well as in the dental lamina (i.e., the epithelial layer connecting the tooth germ to the oral ectoderm, which enables formation of successional teeth in many species; see below) (Brugmann et al., 2007; Liu et al., 2008; Lohi et al., 2010).

Interestingly, *Shh*, initially expressed throughout the oral epithelium, becomes restricted to the early dental placodes (Bitgood and McMahon, 1995; Keränen et al., 1999; Hovorakova et al., 2011). Shh and Wnt/β-catenin signaling appear to closely interact, and several reports advance a downstream position of Shh throughout the different stages of tooth development. Overexpression of the Wnt inhibitor *Dkk1* in the dental epithelium results in downregulation of *Shh* and *Ptch2* expression in developing tooth buds (Liu et al., 2008). Constitutively active Wnt/β-catenin signaling in the epithelium induces (ectopic) *Shh* expression in the tooth germs (Järvinen et al., 2006; Liu et al., 2008; Wang et al., 2009). Recently, TCF/LEF1 binding sites were identified in long-range enhancers of *Shh* that are active in the oral cavity during development, strongly suggesting that Wnt/β-catenin signaling can directly regulate *Shh* expression during odontogenesis (Sagai et al., 2009, 2017; Seo et al., 2018). It remains to be investigated whether or not *Wnt3* and *Wnt7b* inhibit *Shh* expression, leading to its extinction in the non-placodal region and resultant specific presence in the prospective dental placode. More in general, tooth phenotypes in *Wnt3* and *Wnt7b* knockout models have not yet been described (van Amerongen and Berns, 2006). It would be highly valuable to more finely resolve the expression patterns of *Shh* and *Wnt* genes during tooth initiation in a granular spatio-temporally manner [e.g., *via* single-cell (sc) spatial transcriptomics; (Marx, 2021)]. Moreover, inferring cell-cell communication through specific Wnt ligand-receptor interactions using bioinformatical tools [such as CellPhoneDB or NicheNet; (Browaeys et al., 2020; Efremova et al., 2020)] to decipher which cells can selectively respond to distinct Wnt ligands, would generate novel insights in Wnt action specificity during odontogenesis, and in how these precise interactions may play a role in specifying the prospective sites of tooth development.

In addition to Wnt and Shh, *Fgf8*, also one of the first markers of tooth initiation (Neubüser et al., 1997; Keränen et al., 1999), is expressed in a posteriorly located rosette of oral epithelial cells (Figure 2) (Prochazka et al., 2015). Through genetic lineage tracing, cell ablation and live imaging, it was found that a small group of *Shh*-expressing cells triggers these *Fgf8*-expressing epithelial rosettes to migrate toward the initiating tooth bud, where they contribute essential cell mass for tooth development (Prochazka et al., 2015). Moreover, FGF signaling is found necessary, as well as sufficient, to induce subsequent, proliferation-dependent stratification of the dental placode (Li et al., 2016).

The oral ectoderm’s odontogenic band is not only characterized by *Wnt10b* and *Shh* expression, but also by the presence of pituitary homeobox 2 (*Pitx2*). Together, they form the earliest known signals of tooth initiation (Figure 2). Moreover, it has recently been suggested that PITX2 acts upstream of *Shh* expression and formation of the IK signaling center for the placode-to-bud transition (Yu W. et al., 2020). PITX2 may directly activate *Shh* expression *via* a consensus PITX2-binding motif upstream of the *Shh* transcription start site (Yu W. et al., 2020). In addition, PITX2 has been shown to regulate *Lef1* and *Sox2* expression, as well as IK and EK markers such as *Cdkn1a, Shh*, and *Wnt10b* (Ahtiainen et al., 2016; Yu W. et al., 2020). *Lef1*-expressing cells are present in the anterior part of the odontogenic band and SOX2-expressing cells in the posterior zone (St.Amand et al., 2000; Sun et al., 2016; Sanz-Navarro et al., 2018). These SOX2^+^ cells constitute the early progenitor cells of the various epithelial lineages that arise during odontogenesis, and the *Lef1*^+^ cells generate the IK and EK signaling centers (Sun et al., 2016; Sanz-Navarro et al., 2018). The importance of PITX2 in regulating the expression of key signaling center genes is corroborated by the fact that in *Pitx2*-deficient dental epithelium both IK and EK fail to develop (Yu W. et al., 2020).

### Wnt and Shh Signaling Play a Crucial Role in Placode-to-Bud Transition

*Shh* is known to play a key role in the transition from dental placode to bud stage (Figure 1), by coordinating cellular dynamics required for epithelium invagination into the underlying mesenchyme [reviewed in Kim et al. (2017), Seppala et al. (2017), and Hosoya et al. (2020b)] (Li et al., 2016). Inhibition of Shh signaling with cyclopamine annihilates this invagination, thereby impairing growth of the tooth buds (Li et al., 2016). Wnt/β-catenin signaling is also required for bud formation since inhibition of epithelial Wnt/β-catenin through *Dkk1* overexpression prevents the development of this stage (Li et al., 2020). Moreover, the finding that *Shh*, *Lef1* and *Wnt10b* expression largely coincides with the IK supports their essential role in IK regulatory signaling during this early transition phase (Ahtiainen et al., 2016; Yu W. et al., 2020; Mogollón et al., 2021).

Recently, a novel concept of epithelial cell migration during early invagination of ectodermal placodes such as teeth has been described (Li et al., 2016, 2020; Panousopoulou and Green, 2016; Kim et al., 2017). First, coordinated vertical cell movements occur (designated as ‘vertical telescoping’) (Li et al., 2020). Then, suprabasal cells (in the ‘canopy’) horizontally intercalate and migrate centripetally (dubbed ‘canopy contraction’) providing a further tensile stimulus for invagination of the placode (Li et al., 2016, 2020; Panousopoulou and Green, 2016). The latter process is Shh-dependent, as application of cyclopamine inhibits this process (Li et al., 2016, 2020). Although not formally shown yet, Wnt/β-catenin signaling is also expected to play a key role in regulating these epithelial cell dynamics since impaired Wnt/β-catenin signaling prevents bud morphogenesis and formation (Andl et al., 2002; Liu et al., 2008; Panousopoulou and Green, 2016; Li et al., 2020). Both canopy contraction and vertical telescoping rely on an intact F-actin cytoskeleton, and canopy contraction depends on E-cadherin which transmits tensile forces through epithelia (Orsulic et al., 1999; Nelson and Nusse, 2004). β-catenin is known to mediate the structural organization and function of cadherins (at adherens junctions between cells) by linking them to the actin skeleton *via* α-catenin, and E-cadherin can reduce the availability of β-catenin by sequestering it at the plasma membrane (Orsulic et al., 1999; Nelson and Nusse, 2004). Moreover, Wnt/β-catenin signaling can directly modulate E-cadherin (*Cdh1*) transcription *via* a TCF/LEF1 binding site in the *Cdh1* gene promoter (Marin-Riera et al., 2018; Yamada et al., 2019). Together, Wnt/β-catenin is linked to central players in cell movement. Moreover, although outside the scope of this review, it is important to note that non-canonical Wnt signaling, which is strongly intertwined with cell morphology and cytoskeletal remodeling, is also likely to be involved in these processes.

### Wnt/β-Catenin Signaling in the Early Dental Mesenchyme Appears Tightly Regulated

While the Wnt/β-catenin pathway is activated in the epithelium of the dental placode, Wnt antagonists are upregulated in the dental mesenchyme, and mesenchymal Wnt/β-catenin activity is accordingly reduced (Sarkar and Sharpe, 1999; Järvinen et al., 2018), suggesting that this downregulation is needed for proper odontogenesis. In line, constitutively active β-catenin in dental mesenchyme impairs tooth germ morphogenesis (Chen et al., 2019), and elevated β-catenin activation *in vitro* in incisor re-aggregates of dissociated primary dental epithelial and mesenchymal cells is detrimental to tooth formation (Liu et al., 2013). However, full deletion of β-catenin in the dental mesenchyme also arrests tooth development in the bud stage (Chen et al., 2009; Fujimori et al., 2010), and constitutively active β-catenin in palatal mesenchyme induces ectopic tooth bud-like invaginations (Chen et al., 2009). Hence, it is clear that Wnt/β-catenin activity in the early dental mesenchyme needs to be strictly fine-tuned for correct odontogenesis.

Genetic abrogation of mesenchymal β-catenin results in decreased expression of *Bmp4* in the dental papilla (Fujimori et al., 2010), a well characterized mesenchymal-to-epithelial signaling molecule during odontogenesis (O’Connell et al., 2012). *Bmp4* is both a target gene of β-catenin/TCF1/LEF1 complexes, as well as a positive regulator of *Lef1* expression, thus generating a positive Wnt-BMP feedback loop (Chen et al., 1996; Fujimori et al., 2010). A consequence of deficient mesenchymal BMP4 signaling is a decrease in *Shh* expression in the adjacent dental epithelium, suggesting that BMP4 is necessary for the maintenance of *Shh* expression (Zhang et al., 2000; Fujimori et al., 2010). Along the same line, constitutive activation of mesenchymal β-catenin results in increased expression of the BMP antagonist noggin in the dental epithelium resulting in reduced epithelial *Shh* expression (Chen et al., 2019). A similar effect was observed in *Bmp4*-deficient mice, or in *ex vivo* tooth germ explants exposed to noggin-soaked beads (Fujimori et al., 2010; Munne et al., 2010).

Taken together, during the earliest stages of tooth initiation and morphogenesis, Wnt and Shh signaling appear crucially intertwined, both in a direct and an indirect manner.

### Wnt/β-Catenin and Shh Signaling Are Strictly Controlled During Bud and Cap Stage

The pEK produces Wnts, Shh, BMPs, and FGFs that promote adjacent dental epithelium to enwrap the underlying dental papilla and form the typical cap shape (Ahrens, 1913; Jernvall et al., 1994, 1998; Vaahtokari et al., 1996; Thesleff and Sharpe, 1997). The bud-to-cap transition appears mainly proliferation-independent but driven by cytoskeletal remodeling (Yamada et al., 2019). The process is disrupted when focal adhesion kinase (FAK), a key player in epithelium bending, is pharmacologically inhibited (Yamada et al., 2019). Intriguingly, in the epithelial morphogenetic processes of zebrafish the Wnt pathway (i.e., Wnt5b) appears to collaborate and function upstream of Fak (Gutzman et al., 2018; Hung et al., 2020). FAK has also been linked to Shh signaling in various settings such as cancer cell migration and invasion, and mouse embryonic stem cell cytoskeletal remodeling and motility (Chen et al., 2014; Oh et al., 2018). Further study is required to elucidate the interplay of Wnt, Shh and FAK signaling as drivers of cytoskeletal remodeling during bud-to-cap transition in mouse tooth development.

As mentioned, *Shh*, *Lef1*, and *Wnt10b* expression, as well as β-catenin activity, has not only been found in the IK but also in the pEK (Liu et al., 2008). Again, tight control of Wnt/β-catenin and Shh signaling is crucial for tooth development progression in the pEK-governed stages. Disruption in one of both pathways is associated with defective pEK activity, leading to either impaired tooth formation or supernumerary teeth. Inactivation of *Lef1* halts odontogenesis in the bud stage (van Genderen et al., 1994). Epithelial overexpression of the Wnt antagonist *Dkk1* downregulates *Shh*, *Wnt10b*, and *Lef1* expression, thereby arresting tooth germs in the bud stage (Andl et al., 2002; Liu et al., 2008). Inversely, constitutive activation of dental epithelial β-catenin leads to the formation of supernumerary EKs and resulting teeth (Järvinen et al., 2006; Wang et al., 2009; Xavier et al., 2015). On the other hand, conditional deletion of epithelial *Shh* or *Smo* leads to the development of abnormally small teeth, and fusion of first and second molars, while *Gas1* mutant mice with enhanced Shh signaling activity form supernumerary teeth in the diastemal region (i.e., the zone between molars and incisors) (Dassule et al., 2000; Gritli-Linde et al., 2002; Ohazama et al., 2009). Targeted deletion of suppressor of fused (*Sufu*), a major negative regulator of the Shh pathway, from the dental mesenchyme results in delayed bud-to-cap transition of tooth germs due to a defective pEK (Li et al., 2019). *Sufu* mutants display aberrant *Shh* expression patterns, exhibiting ectopic mesenchymal *Shh* expression and decreased pEK *Shh* expression, suggesting an important role for SUFU in fine-tuning Shh signaling to the specific compartment during bud-to-cap transition. Likely due to decreased epithelial Shh, *Sufu* deletion resulted in defective epithelial cell rearrangements, similar to its proposed role during the placode-to-bud transition (Li et al., 2016, 2019).

### Sostdc1 and Lrp4 Establish a Negative Feedback Loop Between Wnt/β-Catenin and Shh Signaling During the Bud and Cap Stage

**FIGURE 3:**
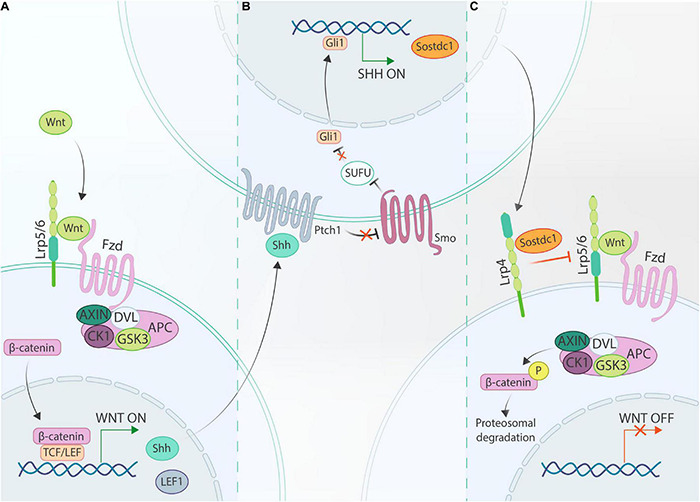
Schematic of the Wnt/Shh/Sostdc1/Lrp4 feedback loop. **(A)** Binding of canonical Wnt ligands to their cognate Fzd receptors, complexed with Lrp5/6 co-receptors, recruits AXIN and DVL to the cell membrane, thus disrupting the β-catenin-degradation complex (consisting of AXIN, DVL, CK1, GSK3, and APC; see text). Consequently, β-catenin can translocate to the nucleus where it complexes with TCF/LEF transcriptional regulators to control expression of Wnt target genes, including *Lef1* and *Shh*. **(B)** In Shh-responsive cells, Shh binds to Ptch receptors (such as Ptch1) thereby relieving Smo inhibition, and as a consequence allowing Smo to inhibit SUFU. This inhibitory action allows for activation of Gli family transcription factors (such as Gli1), which regulate downstream expression of Shh target genes including *Sostdc1*. **(C)** Finally, secreted Sostdc1 cooperates with Lrp4 to inhibit Lrp5/6-dependent Wnt/β-catenin activation.

In recent years, it has been discovered that in developing tooth germs a negative feedback loop is established between Wnt and Shh that is modulated through Sostdc1 and Lrp4, an inhibitory Wnt co-receptor (Figure 3). Joint action of Sostdc1 and Lrp4 in this network was first exposed by the finding that *Lrp4* mutant mice phenocopy *Sostdc1* mutants, i.e., generating fused molars and supernumerary incisors and molars (Kassai et al., 2005; Yanagita et al., 2006; Ohazama et al., 2008; Munne et al., 2009; Ahn et al., 2010). This phenotype resembles the one of genetically modified mice with constitutively active β-catenin or *Gas1* null mutation (which leads to enhanced Shh signaling) (Järvinen et al., 2006; Ohazama et al., 2009; Wang et al., 2009). Secondly, Sostdc1 protein was found to bind Lrp4 using immunoprecipitation analysis (Ohazama et al., 2008). Moreover, *Shh*^±^*;Sostdc1*^±^ mice display elevated Wnt signaling compared to *Sostdc1*^±^ mice, further strengthening the presence of this interactive network, and in particular of the negative feedback loop between Shh and Wnt through Sostdc1 (Ahn et al., 2010; Cho et al., 2011). Furthermore, (epithelial-derived) Shh can directly induce expression of *Sostdc1* in the dental mesenchyme, whereas *in vivo* suppression of Shh signaling using an anti-Shh neutralizing antibody reduces *Sostdc1* expression levels (Cho et al., 2011; Kim et al., 2019). All findings together materialize the model that Shh, which itself is a Wnt target gene, negatively modulates Wnt/β-catenin signaling through its target gene *Sostdc1* which by binding to Lrp4 acts in concert with this inhibitory Wnt co-receptor (Figure 3) (Ohazama et al., 2008). Through elegant combinations of genetic mouse models (such as *Sostdc1, Lrp4, Lrp5*, *Lrp6*, *Shh*, and *Ptch1* full or heterozygous knockouts) exhibiting differing dosages of Shh and/or Wnt/β-catenin signaling, it was established that a properly balanced Shh and Wnt/β-catenin activity is paramount (Ahn et al., 2017). If one of both pathways is completely disrupted, the balance is not maintained, resulting in either impaired tooth growth, or development of supernumerary teeth. This model is consistent with previous findings from *Lef1*, *Smo*, *Gas1*, and *Lrp4* null mice (van Genderen et al., 1994; Gritli-Linde et al., 2002; Ohazama et al., 2008, 2009). Crossing *Lrp4^–/–^* with *Sostdc1^–/–^* mice revealed that *Lrp4* deletion remedied the *Sostdc1^–/–^* phenotype. Even more, gain-of-function *Sostdc1* mutants do not display a phenotype in *Lrp4^–/–^* tooth germs. Of note, genetically reduced *Lrp5* or *Lrp6* dosage appeared to ameliorate the *Lrp4^–/–^* phenotype.

The proposed negative feedback model (Figure 3) appears also true in other ectodermal appendages (such as vibrissae, hair follicles and mammary glands) (Lee et al., 2011; Närhi et al., 2012; Ahn et al., 2013), and thus may represent a conserved regulatory mechanism. Of note, Shh stimulates *Sostdc1* expression predominantly in the dental mesenchyme, which in turn signals back to the epithelium, thus presenting a prime example of the epithelial-mesenchymal crosstalk during odontogenesis. Interestingly, *Sostdc1* expression has also been detected in the incisor mesenchyme before the bud stage as early as E12 (Kim et al., 2019), suggesting that this feedback loop may already play a role in early placode development, although at present not demonstrated. On the other hand, recent data have indicated that the interactive loop remains crucial in later developmental stages such as during cusp patterning (Cho et al., 2011; Ahn et al., 2017).

**FIGURE 4:**
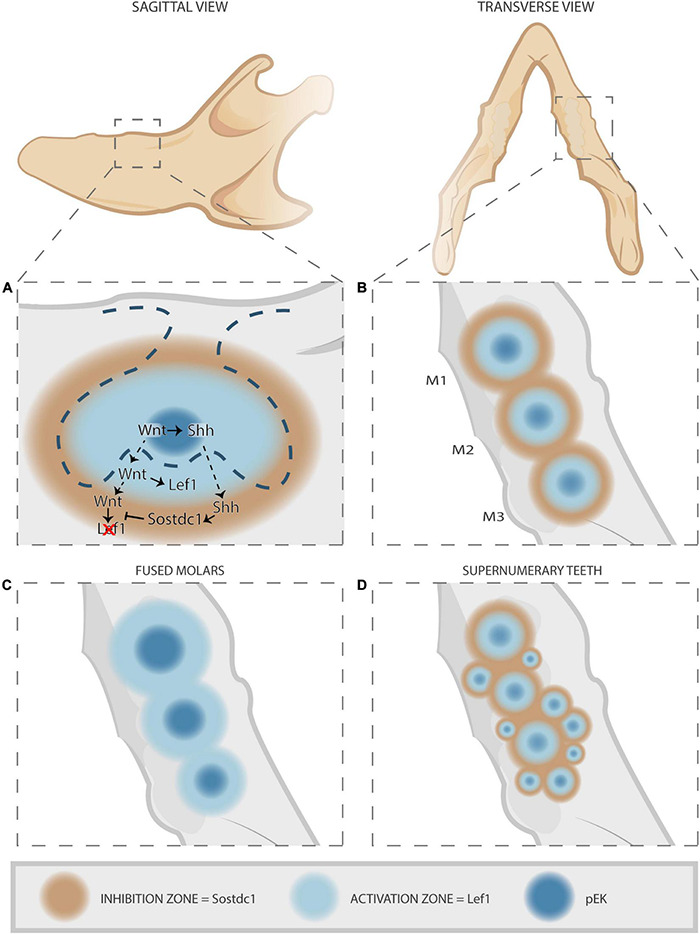
Establishment of tooth-forming and -inhibiting zones by the Wnt/Shh/Sostdc1/Lrp4 feedback loop, and consequences of disruption. **(A)** Sagittal view of developing tooth germ. Short-range Wnt signals from the pEK (dark blue) induce localized *Shh* expression. Together, Wnt and Shh diffuse laterally into the surrounding dental epithelium and mesenchyme, inducing expression of their target genes *Lef1* and *Sostdc1*, respectively. Due to short-reaching Wnt and farther-reaching Shh diffusion, mutually exclusive domains of either *Lef1* or *Sostdc1* expression are established. This pattern creates a tooth-forming activation zone (light blue) and an inhibition zone where tooth formation is prevented (brown). **(B)** Transverse view of developing tooth germs. In correctly developing teeth, the established tooth formation and inhibition zones allow proper development of sequential teeth (M, molars). **(C,D)** When disrupted, the regulatory loop fails to establish the inhibition zone leading to fused teeth **(C)**, or alternatively excess activation and inhibition zones are formed yielding supernumerary teeth **(D)**. Figure inspired by Cho et al. (2011).

Upon disruption of the Wnt/Shh/Sostdc1/Lrp4 loop, typical tooth patterning aberrations occur resulting in either fused molars or supernumerary molars (Figures 3, 4) and incisors. Supernumerary teeth can be the consequence of sustained survival and development of rudimentary (vestigial) tooth germs, or of expansion and/or splitting of the pEK signaling center. Indeed, *Lrp4* and *Sostdc1* null mutants (thus, showing elevated Wnt signaling) display sustained development of R2, a rudimentary tooth bud located distally from the first molar in the diastema (Ahn et al., 2010, 2017). Moreover, based on genetic and spatial-expression analyses of feedback loop components, the regulatory mechanism has been proposed to control spatial patterning of dentition in the bud stage (Figure 4) (Cho et al., 2011). Short-acting Wnt signals in the pEK induce *Shh* production from the pEK (Figures 3, 4A). Wnt and Shh diffuse laterally into the surrounding epithelium and mesenchyme, inducing expression of the target genes *Lef1* and *Sostdc1*. Their expression appears mutually exclusive, with the *Lef1*-expressing domain closest to the pEK and surrounded by a broader *Sostdc1*-expressing domain. This creates a tooth-forming activation and inhibition zone, respectively, thus preventing fusion of adjacent developing tooth germs (Figures 4A,B). If this regulatory loop is disrupted, the inhibition zone may not be established, resulting in fused teeth (Figure 4C). Alternatively, an excess number of activation and inhibition zones may be formed, generating supernumerary teeth (Figure 4D). Given that *Sostdc1* is mainly a mesenchymal signal, the model supports a role for the dental mesenchyme in controlling the number of teeth during development. In line, earlier work reported that removal of mesenchymal tissue from incisor explants resulted in formation of *de novo* incisors, similar to the phenotype in *Sostdc1* deficiency (Munne et al., 2009).

Of final note here, the Wnt/Shh/Sostdc1/Lrp4 regulatory loop may be tightly intertwined with the above described Wnt/BMP positive feedback loop (which can modulate *Shh* expression) during prior developmental stages (Zhang et al., 2000; Fujimori et al., 2010). In addition to its nature as Wnt antagonist, Sostdc1 is also able to bind BMP ligands with high affinity to act as a BMP antagonist (Laurikkala et al., 2003; Yanagita et al., 2004). Furthermore, BMPs can induce expression of *Sostdc1* in pEK (Laurikkala et al., 2003).

### Crown Development: Formation of Molar Cusps Is Governed by Inductive Wnt/β-Catenin and Inhibitory Shh/Sostdc1 Signaling

**FIGURE 5:**
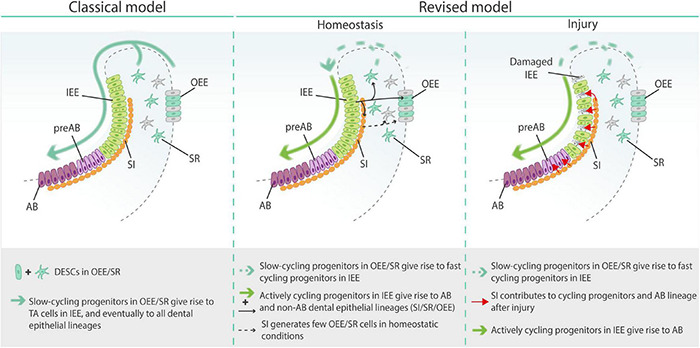
Classical and revised models of epithelial stem/progenitor cells in the mouse tooth. AB, ameloblasts; DESCs, dental epithelial stem cells; IEE, inner enamel epithelium; OEE, outer enamel epithelium; preAB, pre-ameloblasts; SI, stratum intermedium; SR, stellate reticulum; TA cells, transit-amplifying cells.

The dental epithelium continues to burrow into the underlying mesenchyme, eventually enwrapping the condensed dental papilla, thereby successively forming the typical cap and bell shapes (Figure 1A). The IEE, SR, stratum intermedium (SI, a layer of non-ameloblast dental epithelial cells lying beneath both ameloblasts and IEE; see Figure 5) and OEE morphogenetically form in the continuously growing tooth germ. Like in the earlier stages of odontogenesis, interplay and strict levels of Wnt/β-catenin and Shh signaling are paramount in these processes.

As mentioned, the pEK undergoes apoptosis once the cap stage is reached, but in multicuspid molars, some pEK cells escape their demise and contribute to sEK formation (Du et al., 2017). sEKs display hallmarks identical to IKs and pEKs, including a non-proliferative G1-phase state with expression of *Cdkn1a*, *Wnt10b*, *β-catenin*, *Axin2*, and *Shh* (Sarkar and Sharpe, 1999; Liu et al., 2008; Lohi et al., 2010). sEKs are localized at the tips of the prospective cusps and guide the ensuing cusp formation. In accordance with pEK defects due to malfunctioning of Wnt/β-catenin signaling, inhibition of the pathway at the bell stage (E16) via *Dkk1* overexpression results in disrupted sEKs and impaired cusp development, culminating in blunted, less protruding cusps (Liu et al., 2008). In contrast, suppression of *Shh* (via antibody treatment) or deletion of *Sostdc1* narrows the intercuspal distance and results in supernumerary cusp structures (Kassai et al., 2005; Cho et al., 2011; Kim et al., 2019).

Together, these findings support the hypothesis that the Wnt/Shh/Sostdc1 regulatory loop remains in play during the later stages of tooth development. The cusp defects strongly resemble the earlier tooth patterning aberrations. Analogous to their activities in tooth patterning, Wnt/β-catenin appears to be a driver in cusp formation, while Shh/Sostdc1 inhibits cusp development.

### Dental Cytodifferentiation

#### Development of Ameloblasts and Odontoblasts

Once the tooth germ shape is fully established at the bell stage (E18.5), enamel-producing ameloblasts start to differentiate from the IEE and dentin-generating odontoblasts from the adjacent dental papillary cells, both separated by a basement membrane, through an active interplay. The ameloblast lifecycle consists of four stages, defined by morphological and functional hallmarks.

In the first (presecretory) stage, IEE cells (pre-ameloblasts) initiate the cytodifferentiation process by inducing progression of adjacent dental papilla into odontoblasts which deposit a fine layer of pre-dentin at the future dentino-enamel junction (DEJ; Figure 1A) (Bei, 2009; Bartlett, 2013). Pre-dentin, primarily composed of collagen, reciprocally instructs pre-ameloblasts to differentiate into secretory ameloblasts. Meanwhile, actively secreting odontoblasts form large columnar cells with processes extending into the (pre-)dentin. During the differentiation progression, pre-ameloblasts break through the basement membrane at the DEJ, elongate from short cuboidal into tall columnar cells, and form so-called Tomes’ processes at their apical enamel-forming ends.

During the second episode, secretory-stage ameloblasts start to secrete enamel matrix proteins such as amelogenin, enamelin, and ameloblastin to form a protein-rich and soft enamel matrix. Ameloblasts are also equipped with a variety of mineral and bicarbonate transporters to drive mineral growth within the matrix (Bronckers, 2017). In addition, secretory-stage ameloblasts produce matrix metalloproteinase 20 (MMP20), which hydrolyses enamel matrix proteins into stable intermediates (Bartlett et al., 1998; Fukae et al., 1998). Within this protein matrix, each ameloblast forms a thin enamel rod or enamel crystallite. As the enamel thickens, ameloblasts move away from the dentin surface, and the crystallites grow longitudinally, parallel to each other. In addition to moving backward, groups of ameloblasts slide past each other forming the typical decussate pattern of rodent enamel (Reith and Ross, 1973). At the end of the secretory stage, the enamel layer reaches its full thickness.

In the third (transition) stage, ameloblasts retract their Tomes’ processes, contract again and deposit a new basal lamina. Ultimately, in the fourth and final (maturation) stage, ameloblasts cycle between two morphologies at the enamel surface: ruffle-ended and smooth-ended. The ameloblasts degrade and reabsorb the enamel protein matrix through secretion of proteolytic enzymes [e.g., kallikrein-related peptidase-4 (KLK4)], while the enamel crystallites continue to grow and expand. Eventually, the fully formed enamel is highly mineralized with very little amount of protein remaining, thereby forming the hardest substance in the body.

#### Cell-Autonomous Wnt/β-Catenin Signaling Is Crucial in Both Odontoblast and Ameloblast Development During Crown Formation

##### Odontoblast development

To study the role of Wnt during dental cytodifferentiation, several studies have employed cell-type specific knockout of Wntless (*Wls*), a chaperone protein required for proper cellular secretion of Wnt ligands (Bänziger et al., 2006; Bartscherer et al., 2006). Targeted *Wls* disruption in developing odontoblasts highlighted that cell-autonomous Wnt is crucial for proper odontoblast differentiation and dentin formation (dentinogenesis), as well as for tooth root development (see below) (Lim et al., 2014; Bae et al., 2015). In analogy, odontoblast-specific overexpression of *Dkk1* or deletion of β-catenin (Han et al., 2011; Kim et al., 2013) resulted in identical phenotypes, whereas constitutive activation of β-catenin in the dental mesenchyme induced premature odontoblast differentiation and excessive dentin and cementum materialization (Kim et al., 2011). Moreover, the promotor regions of collagen type Iα1 (*Col1a1*, the key component of dentin) and decorin (*Dcn*, a proteoglycan component of dentin) contain TCF/LEF binding sites and have been identified as Wnt/β-catenin target genes during odontoblast differentiation (Guan et al., 2016).

Interestingly, Wnt activation by ubiquitous genetic deletion of the secreted Wnt inhibitor *Notum* severely disrupted formation of both crown and root dentin in molars and of crown dentin in incisors, but did not affect amelogenesis (Vogel et al., 2016). In incisors, NOTUM was recently found to be associated with early odontoblasts localized near the CL mesenchymal area, although its precise role during odontoblast differentiation remains to be elucidated (Krivanek et al., 2020). Similarly, ablation of mothers against decapentaplegic homolog 4 (*Smad4*), a key regulator of transforming growth factor beta (TGFβ)/BMP signaling, in the dental mesenchyme, resulting in downregulation of the Wnt inhibitors *Dkk1* and *Sfrp1* and thus Wnt pathway activation, caused impaired odontoblast differentiation and dentinogenesis, but had no effect on ameloblast differentiation (Li et al., 2011). Moreover, this Wnt signaling boost through *Smad4* inactivation resulted in a switch from dentinogenesis to osteogenesis, indicating the importance of finely balanced Wnt/β-catenin activity in determining the cell fate of the dental mesenchyme. Together, Wnt/β-catenin in the dental mesenchyme is cell-autonomously required for proper odontoblast differentiation during crown (and root) development.

##### Ameloblast development

A similar cell-autonomous role of Wnt/β-catenin has been identified in ameloblast differentiation. Deletion of *Wls* in the *Shh*-expressing dental epithelial lineage leads to defective ameloblast development, suggested to be largely due to decreased expression of Shh pathway components such as *Shh*, *Gli1*, and *Ptch1* (Xiong et al., 2019). Epithelial deletion of β-catenin resulted in enamel defects including disorganized ameloblasts which lacked the typical columnar shape, and dysplastic, soft enamel (Yang et al., 2013; Guan et al., 2016). Furthermore, pharmacological β-catenin inhibition showed impaired migration and invasion of ameloblast lineage cells *in vitro* (Guan et al., 2016). Overexpression of *Wnt3* in the dental epithelium resulted in disorganized ameloblasts and enamel defects (Millar et al., 2003). In analogy, pharmacological inhibition of GSK3β, leading to β-catenin activation, impaired ameloblast differentiation and cell polarity in mouse tooth explant cultures (Aurrekoetxea et al., 2016; Yang et al., 2018). Moreover, constitutive activation of β-catenin in ameloblasts led to severely disorganized ameloblasts lacking their normal polarity, as well as to chalky hypoplastic enamel (Fan et al., 2018). Although enamel thickness was not significantly reduced, its mineralization was delayed, and decreased expression of *Mmp20* and *Klk4* resulted in failure of matrix protein removal. Of note, deletion of *Wls* specifically in the dental epithelium also caused impaired differentiation of the mesenchymal odontoblasts (Xiong et al., 2019).

Overexpression or ablation of *Mmp20* resulted in enamel defects and aberrant ameloblast behavior in both molars and incisors (Bartlett et al., 2011; Shin et al., 2016, 2018). The phenotype was characterized by dysplastic enamel, disruption of the ameloblasts’ layer and morphology, infiltration of ameloblasts in the enamel space (in incisors) or in the underlying SI and SR cell layers (in molars), and presence of ectopic mineralization nodules (Shin et al., 2016, 2018). In addition to cleaving enamel matrix proteins, MMP20 plays an important role in ameloblast cell morphology and motility. Changes in morphology and increased migration/invasion in the case of *Mmp20*-overexpressing ameloblasts have been attributed to enhanced nuclear localization of β-catenin and signaling, since prevented by *in vitro* pharmacological inhibition of β-catenin (Shin et al., 2018). MMP20 cleaves cadherin extracellular domains (Bartlett et al., 2011; Guan and Guan and Bartlett, 2013). Consequently, MMP20 overexpression leads to increased cleavage of E-cadherin resulting in release of β-catenin from the cell membrane which can translocate to the nucleus.

Finally, ChIP-Seq experiments have identified β-catenin and transcription factor 7 like 2 (TCF7L2, also known as TCF4) binding regions in the *Mmp20* promoter, at least in human colon cancer cells (Bottomly et al., 2010). Constitutive activation of β-catenin in ameloblasts results in decreased levels of MMP20 (Fan et al., 2018). Thus, a feedback loop may exist between Wnt/β-catenin and MMP20. Moreover, MMP20 may also interact with Shh, given common roles in cell migration. Numerous studies have identified other MMPs [such as MMP2 and MMP9 as targets of Shh signaling (Bigelow et al., 2005; Yoo et al., 2011; Chen et al., 2013; Fan et al., 2014)], and *Mmp20* expression may also be regulated by Shh. Nevertheless, a direct relationship of MMP20 with Wnt/β-catenin or Hh signaling in mouse tooth development remains to be conclusively defined.

### Wnt and Shh During Tooth Root Development

#### Tooth Root Formation: Involvement of Hertwig’s Epithelial Root Sheath

Different from the tooth crown, which is shielded by enamel, tooth root is covered by the much softer cementum. Tooth root development starts once the crown is formed, i.e., when the enamel reaches the future junction with the cementum layer [cemento-enamel junction (CEJ)], the prospective boundary between crown and root (Li et al., 2017) (Figure 1B). The CL-derived HERS (Figure 1A) extends apically to guide root formation, determining the number, shape and size of the roots (Ten Cate, 1996; Li et al., 2015, 2017; Yu and Klein, 2020). Where inner layer HERS cells contact apical papilla mesenchymal cells, the latter differentiate into odontoblasts which deposit root dentin. Approximately 1 week after its apical elongation, HERS disintegrates, thereby giving rise to a structure resembling a mesh or fishnet (Huang et al., 2009). This perforated HERS network allows dental follicle cells to pass through and contact the papillary mesenchyme and the root dentin (Luan et al., 2006; Huang et al., 2009). In a manner similar to ameloblast differentiation, contact with deposited dentin allows dental follicle cells to differentiate into cementum-producing cementoblasts (Luan et al., 2006; Zeichner-David, 2006; Huang et al., 2009; Li et al., 2017).

In parallel, HERS cells can undergo epithelial-to-mesenchymal transition (EMT) and directly contribute to the cementoblast population (Huang et al., 2009). Importantly, HERS plays a crucial role in the formation of the PDL, both *via* direct contribution to the PDL lineage after undergoing EMT and secretion of signaling molecules (Itaya et al., 2017; Li et al., 2017). Some dental follicle cells adjacent to HERS migrate in between the root and the alveolar bone, forming PDL fibroblasts (Xiong et al., 2013; Li et al., 2017). These cells secrete collagen fibers, that will eventually become thick, highly organized bundles termed Sharpey’s fibers (Xiong et al., 2013; Li et al., 2017). The fibers are embedded in the cementum and connect to the alveolar bone, helping the tooth to remain embedded in the jaw and resist the forces generated during, among others, mastication. Eventually, the remaining HERS fragments become dispersed throughout the dental follicle thereby establishing the ERM, a quiescent epithelial cell population that has been taxed with stem cell properties and can contribute to cementum and PDL regeneration and repair (Huang et al., 2009; Takahashi et al., 2019).

Developing tooth roots display high levels of Wnt/β-catenin activity as shown by *Axin2* expression, which is strongly associated with HERS, surrounding apical dental papilla, root odontoblasts and cementocytes (Lohi et al., 2010; Choi et al., 2020). Also during this phase of tooth development, Wnt/β-catenin is critical in both the epithelial- and mesenchymal-derived compartments for root dentinogenesis as well as cementogenesis.

#### Elevation of Wnt/β-Catenin Activity Is Crucial to Shift Dental Epithelium From Crown Toward Root Fate

During the crown-to-root transition, the dental epithelial cells, generating HERS, contribute to root formation and cementogenesis instead of amelogenesis. As during the latter process, the TGFβ/BMP/Wnt/β-catenin feedback loop plays an important role to fine-tune Wnt/β-catenin activity. Indeed, the transition is characterized by a reduction in epithelial TGFβ/BMP signaling leading to elevated Wnt/β-catenin activity, needed to shift the dental epithelium cell fate from crown to root lineage (Yang et al., 2013; Li et al., 2015). Disruptions in the TGFβ/BMP pathway, for instance *via* deletion of epithelial *Smad4* or BMP receptor 1A (*Bmpr1a*, also known as *Alk3*), can severely impact crown/root development (Yang et al., 2013; Li et al., 2015). Precocious decline of epithelial TGFβ/BMP signaling (i.e., during crown formation instead of during the crown-to-root transition) by *Bmpr1a* ablation shifts the dental epithelial cell fate toward HERS/cementoblast lineage and results in both ectopic cementum and HERS/ERM in the enamel region, as well as increased production of orthotopic cementum (Yang et al., 2013). Additional deletion of β-catenin rescues this phenotype and prevents precocious cementogenesis, further strengthening the idea that reduction of TGFβ/BMP signaling is necessary to elevate Wnt/β-catenin to advance root development. In analogy, implantation of BMP4-soaked beads near HERS prevented its elongation and proliferation (Hosoya et al., 2008). In a mouse model with strongly elevated cementogenesis [i.e., mutant in ectonucleotide pyrophosphatase/phosphodiesterase family member 1 (*Enpp1*), a key regulator of extracellular diphosphate levels in mineralizing tissues], β-catenin and TCF/LEF activity is highly increased, thus reinforcing Wnt/β-catenin’s key role in this process (Choi et al., 2019). Stabilization of β-catenin specifically in the dental mesenchyme during cementogenesis results in excessive cementum formation, in line with the potential of dental follicle cells to generate cementoblasts, likely driven by Wnt/β-catenin (Kim et al., 2011).

Recent studies further emphasized the crucial role of epithelial Wnt/β-catenin signaling during root development. Through various *Wnt10a* knockout models, it was demonstrated that epithelial Wnt10a is required for proper formation of the tooth root (Xu et al., 2017; Yu M. et al., 2020). General deletion of *Wnt10a in* epithelial, but not mesenchymal cells, resulted in a typical taurodont phenotype (i.e., with elongated root trunks and lack of root furcation), similar to the phenotype observed in humans with *WNT10a* mutations (see below, Table 1) (Yang et al., 2015; Xu et al., 2017; Yu M. et al., 2020). Epithelial-derived Wnt10a was found crucial for the proliferation of the epithelium, and thus the extension of the HERS. In contrast, the epithelial Wnt10a inhibited the proliferation of mesenchymal cells, likely *via* inhibition of mesenchymal Wnt4 production (Yu M. et al., 2020). The necessity of Wnt/β-catenin for HERS elongation was further confirmed in a recent study applying conditional β-catenin deletion in *Shh*^+^ HERS, leading to disruption of both cell proliferation and dynamics (Yang et al., 2021). Eventually, *Wnt10a*, which appears initially expressed in both HERS and dental papilla, gradually becomes restricted to the mesenchymal cells in the dental papilla and dental follicle, and to the odontoblasts (see below) (Yu M. et al., 2020).

**TABLE 1:** Wnt and Shh pathway components mutated in human diseases with dental pathology.

**Gene mutated**	**Pathway effect**	**Disease**	**References**
APC	Wnt hyperactivation	- Gardner syndrome, Familial Adenomatous Polyposis 1 (MIM 175100)	Half et al., 2009; Siriwardena et al., 2009
		- Odontogenic carcinoma.	
AXIN2	Wnt hyperactivation	Familial, non-syndromic hypodontia/tooth agenesis	Lammi et al., 2004; Mostowska et al., 2006; Yu et al., 2019
CTNNB1	Wnt hyperactivation	- Ameloblastoma.	Sekine et al., 2002, 2003; Kawabata et al., 2005; Siriwardena et al., 2009; Brown et al., 2014; Yukimori et al., 2017
		- Odontogenic carcinoma.	
		- Calcifying odontogenic cysts.	
		- Adamantinomatous craniopharyngioma.	
DKK1	Wnt hyperactivation	Familial, non-syndromic tooth agenesis	Liu et al., 2012; Dinckan et al., 2018; Yu et al., 2019
DVL1	Wnt inactivation	Robinow syndrome, autosomal dominant 2 (MIM 616331)	White et al., 2015
DVL3	Wnt inactivation	Robinow syndrome, autosomal dominant 3 (MIM 616894)	White et al., 2016
FZD2	Wnt inactivation (non-canonical)	Omodysplasia 2 (MIM 164745); Robinow syndrome-like	Nagasaki et al., 2018; White et al., 2018
GLI2	Shh inactivation	Holoprosencephaly (9) with solitary median maxillary incisor (MIM 610829)	Roessler et al., 2003
GLI3	Shh inactivation	Familial, non-syndromic hypodontia/tooth agenesis	Liu et al., 2012
KREMEN1	Wnt hyperactivation	Ectodermal dysplasia 13, hair/tooth type (MIM 617392)	Issa et al., 2016; Dinckan et al., 2018
LRP4	Wnt hyperactivation	Cenani-Lenz syndrome with dental anomalies (MIM 212780)	Li et al., 2010
LRP6	Wnt inhibition	Familial, non-syndromic tooth agenesis	Massink et al., 2015; Dinckan et al., 2018; Yu et al., 2019
NXN	Wnt inactivation (non-canonical)	Robinow syndrome, autosomal recessive 2 (MIM 618529)	White et al., 2018
PORCN	Wnt inhibition	Focal Dermal Hypoplasia (MIM 305600)	Bornholdt et al., 2009; Froyen et al., 2009
PTCH1	Shh activation	- Ameloblastoma. - Basal cell nevus syndrome with odontogenic keratocysts (MIM 601309 and 109400).	Kawabata et al., 2005; Guo et al., 2013; Shimada et al., 2013
PTCH2	Shh activation	Basal cell nevus syndrome with odontogenic keratocysts (MIM 603673 and 109400)	Fujii et al., 2013
ROR2	Wnt inactivation (non-canonical)	Robinow syndrome, autosomal recessive 1 (OMIM 268310)	Afzal et al., 2000; van Bokhoven et al., 2000; Mazzeu et al., 2007
SHH	Shh inhibition	Holoprosencephaly (3) with solitary median maxillary incisor (MIM 147250)	Nanni et al., 2001; Lami et al., 2013
SMO	Shh activation	Ameloblastoma	Brown et al., 2014; Sweeney et al., 2014
SUFU	Shh activation	Basal cell nevus syndrome with odontogenic keratocysts (MIM 607035 and 109400)	Pastorino et al., 2009; Shimada et al., 2013
WNT10A	Wnt inhibition	- Familial, non-syndromic tooth agenesis	Bohring et al., 2009; van den Boogaard et al., 2012; Arte et al., 2013; Song et al., 2014; Dinckan et al., 2018; Yu et al., 2019
		- Odonto-onycho-dermal dysplasia (MIM 257980)	
		- Schöpf-Schulz-Passarge syndrome (MIM 224750)	
WNT10B	Wnt inhibition	Familial, non-syndromic tooth agenesis	Yu et al., 2016, 2019
WNT5A	Wnt inactivation (non-canonical)	Robinow syndrome, autosomal dominant 1 (MIM 180700)	Person et al., 2010; Roifman et al., 2015

#### Non-redundant and Dose-Dependent Role of Shh During Tooth Root Development

Numerous studies have also highlighted the essential and non-redundant role of Shh during tooth root development, interfacing with both Wnt/β-catenin and TGFβ/BMP signaling. Expression of *Shh*, *Ptch1*, and *Gli1* is gradually restricted to the apical IEE and eventually to HERS during crown-to-root transition, while *Ptch1* and *Gli1* are also detected in the surrounding mesenchyme (Nakatomi et al., 2006; Khan et al., 2007). Both constitutive activation and inhibition of the Shh pathway during root development negatively affects cell proliferation and decreases the root length, indicating a strict Shh dose-dependency of tooth root development (Nakatomi et al., 2006; Huang et al., 2010; Li et al., 2015; Liu et al., 2015). Importantly, it has been revealed that, similarly to its role in balancing Wnt/β-catenin activity, TGFβ/BMP signaling also interfaces with the Shh pathway to ensure appropriate levels of Shh activity for proper root development (Huang et al., 2010). Epithelial *Shh* expression is induced by SMAD4 and drives expression of mesenchymal *nuclear factor I/C* (*Nfic*), a key transcription factor during root development (Steele-Perkins et al., 2003; Huang et al., 2010; Kim et al., 2015; Liu et al., 2015). *Nfic* knockout mice display impaired root formation, characterized by reduced cell proliferation, defective odontoblast differentiation and short roots (Steele-Perkins et al., 2003; Kim et al., 2015). Moreover, these mice exhibit elevated Hh pathway activity and *Gli1* expression, indicating a negative feedback loop (Liu et al., 2015). *Nfic* has indeed been shown to interact with the promoter of *Hhip* (Liu et al., 2015) a competitive antagonist of Hh ligands in binding Ptch receptors (Chuang and McMahon, 1999). Further, pharmacologically inhibiting elevated Hh activity in *Nfic^–/–^* mice partially reverses the root phenotype (Liu et al., 2015).

#### Potential Involvement of the Wnt/Shh/Sostdc1 Feedback Loop During Tooth Root Formation and Cementogenesis

Only recently, it has been found that the Wnt/Shh/Sostdc1 regulatory loop may also play a role during the later tooth root development phase. In genetic mouse models with hyperactivated Hh signaling (due to constitutively activated *Smo* or deleted *Sufu* in the mesenchymal compartment), *Sostdc1* (as well as *Dkk1*) expression is elevated and Wnt/β-catenin activity reduced resulting in decreased cementum formation and mineralization (Choi et al., 2020). These defects are rescued in the mutants when β-catenin is constitutively activated, further strengthening the crucial role of tightly regulated Shh as well as Wnt/β-catenin activity during root formation and cementogenesis (Choi et al., 2020). Hence, the Wnt/Shh/Sostdc1 regulatory loop appears to be a conserved feedback mechanism that is repeatedly recycled throughout odontogenesis. Further functional studies of Sostdc1 during root development and cementogenesis remain necessary to validate this hypothesis. Moreover, it would be interesting to explore the role of Lrp4 during these processes, considering its involvement in the Wnt/Shh/Sostdc1 regulatory loop during the prior tooth developmental stages.

#### Interfering With Wnt Signaling Impairs Tooth Root Dentinogenesis

Similarly to their negative impact on crown odontoblast differentiation and dentinogenesis, disruptions in the Wnt/β-catenin pathway, either via overactivation or downregulation, also lead to impaired root odontoblast formation, and to shortened or completely lacking roots (Han et al., 2011; Kim et al., 2013; Lim et al., 2014; Bae et al., 2015; Vogel et al., 2016). Specific deletion of *Wls* in odontoblasts revealed the requirement of cell-autonomous Wnt/β-catenin signaling and production of Wnt ligands not only during crown but also root dentinogenesis (Lim et al., 2014; Bae et al., 2015). For instance, in postnatal day 14 molars, *Wnt10a* is expressed in odontoblasts along the dentin surface as well as in differentiating (pre-)odontoblasts along HERS, where it may be involved in odontoblast differentiation, based on its capacity to activate expression of dentin sialophosphoprotein (*Dspp*), a key non-collagenous component of dentin, as observed *in vitro* in a mesodermal cell line (Yamashiro et al., 2007). Intriguingly, odontoblast differentiation and dentin formation appeared unaffected when *Wnt10a* was genetically deleted, which instead resulted in taurodont teeth (Yang et al., 2015).

Together, these findings highlight the complex roles of the Wnt/β-catenin pathway and its components in odontoblast differentiation and dentinogenesis, and in root furcation and elongation during specific stages of tooth development.

## Dental Epithelial and Mesenchymal Stem Cells: Impact of Wnt/β-Catenin and Shh

### Dental Epithelial Stem Cells: Cellular Origin and Heterogeneity

The origin of dental epithelial stem cells (DESCs) can be traced back to the earliest stages of odontogenesis. During establishment of the dental placodes, the PITX2^+^ odontogenic band of oral ectoderm is subdivided in an anteriorly situated group of LEF1^+^ and a posteriorly situated group of SOX2^+^ cells (St.Amand et al., 2000; Sun et al., 2016; Sanz-Navarro et al., 2018). The SOX2^+^ cells constitute the early progenitors of the various dental epithelium-derived cell lineages, including the enamel-forming ameloblasts, as well as of the later DESCs, both in incisors and molars (Juuri et al., 2012; Li et al., 2015; Sun et al., 2016; Sanz-Navarro et al., 2018). Around E14, *Sox2* expression becomes restricted to the CLs in molars and incisors (in the latter in both liCL and laCL) (Juuri et al., 2012). In the continuously growing mouse incisors, *Sox2* expression eventually disappears from the liCL (∼E15) but strongly persists in the tip of the laCL, where SOX2^+^ stem cells remain present throughout the animal’s life (Juuri et al., 2012; Li et al., 2015). In the mouse molars, SOX2^+^ DESCs are only transiently present in the CL and are lost upon root formation when the molar CL becomes dispersed in HERS and ERM (Li et al., 2015). Throughout the animal kingdom, the presence of SOX2^+^ dental epithelial cells is highly conserved in various mammalian species, not only in sharks, cichlid fish and reptiles where teeth are continuously replaced but also in the non-regenerating human teeth (Fraser et al., 2013; Juuri et al., 2013; Martin et al., 2016; Bloomquist et al., 2019).

At the tip of the CLs, the SR and OEE (Figure 1A) have been suggested to be the location of the true, slow-cycling DESCs, whereas the IEE is considered to contain their progeny, including the proliferating transit-amplifying (TA) cells and the eventual enamel-forming ameloblasts (Kuang-Hsien Hu et al., 2014). *In vivo* lineage tracing has identified DESCs as slow-cycling label [genetic or bromodeoxyuridine (BrdU)]-retaining cells. These studies have uncovered numerous stem cell markers in addition to *Sox2* such as the Shh components *Gli1* (Seidel et al., 2010, 2017; Biehs et al., 2013) and *Ptch1* (Seidel et al., 2010), and the Wnt component and target gene *Lgr5* (Suomalainen and Thesleff, 2010; Chang et al., 2013; Sanz-Navarro et al., 2018), as well as BMI1 proto-oncogene, polycomb ring finger (*Bmi1*) (Biehs et al., 2013), integrin subunit alpha 6 (*Itga6*, also known as *Cd49f*) (Chang et al., 2013), leucine rich repeats and immunoglobulin like domains 1 (*Lrig1*) (Seidel et al., 2017) and insulin like growth factor binding protein 5 (*Igfbp5*) (Seidel et al., 2017). Interestingly, *Gli1* and *Bmi1*, in addition to *Lrig1* and *Igfbp5*, have also been found to distinguish dental epithelial and mesenchymal (see below) stem cell populations (Biehs et al., 2013; Zhao et al., 2014; Seidel et al., 2017).

The DESC population is heterogeneous. Among others, *Lrig1*-expressing cells have been advanced as a subset of *Gli1*-expressing DESCs, and both *Ptch1*- and *Lgr5*-expressing cells have been found to be subsets of *Sox2*-expressing DESCs, with *Lgr5*^+^/*Sox2*^+^ cells representing the smaller subpopulation (Seidel et al., 2017; Sanz-Navarro et al., 2018; Binder et al., 2020). Moreover, these studies have also identified *Lrig1*^+^/*Gli1*^–^, *Ptch1*^+^/*Sox2*^–^ and *Lgr5*^+^/*Sox2*^–^ cell populations. DESC subpopulations do not only display molecular but also functional heterogeneity. For instance, lineage tracing revealed that the progeny of certain DESC subtypes could be rapidly (∼days) detected after tamoxifen induction (e.g., *Sox2*^+^, *Gli1*^+^, and *Bmi1*^+^ DESCs), whereas other subtypes required longer time (∼months) to give rise to descendants of all the epithelial lineages, thus representing more quiescent DESC subtypes (e.g., *Lrig1*^+^ cells) (Seidel et al., 2010).

### Advanced Insights Into Dental Epithelial Stem Cells Heterogeneity From Single-Cell Transcriptomics

This DESC heterogeneity has been confirmed and further expanded by recent sc transcriptomic profiling studies of dental tissues (Sharir et al., 2019; Chiba et al., 2020; Krivanek et al., 2020) [recently reviewed in Fresia et al. (2021)]. Krivanek et al. (2020) confirmed the presence of a heterogeneous DESC compartment expressing multiple previously identified markers (such as *Sox2*, *Lrig1*, and *Lgr5*), including rare *Sox2*^+^/*Lgr5*^+^, *Sox2*^+^/*Lrig1*^+^, and *Sox2*^+^/*Lgr5*^+^/*Lrig1*^+^ subtypes. In addition, the study identified a novel *Acta2*^+^ (actin alpha 2, smooth muscle) stem cell population located predominantly in the outer SR and OEE (Krivanek et al., 2020). Similarly to other DESC markers, *Acta2* was found to be co-expressed with *Sox2* in some cells. Lineage tracing revealed that progeny of ACTA2^+^ cells could be detected in the dental epithelium (following some days) and in mature ameloblasts (after 1–2 months), thus contributing to all dental epithelial lineages (Krivanek et al., 2020). The study also identified novel putative DESC markers (e.g., *Pknox2*, *Zfp273*, *Spock1*, and *Pcp4*), however, still requiring functional validation, as well as a long-lived *Egr*^+^ (early growth response 1)/*Fos*^+^ (Fos proto-oncogene, AP-1 transcription factor subunit)/*Sox2*^–^ progenitor cell population specifically contributing to OEE cells (Krivanek et al., 2020).

Using sc transcriptomics combined with kinetics and trajectory analysis, Sharir et al. (2019) identified a large pool of cycling progenitor cells in the IEE lacking (putative) DESC marker genes (Shadad et al., 2019). The cells display multipotent potential, being capable to differentiate into both ameloblast- and non-ameloblast (SR, OEE, and SI) lineages. This study, as well as the sc transcriptomic profiling by Chiba et al. (2020) in contrast to Krivanek et al. (2020) did not distinguish a uniquely defined, unambiguous stem cell (DESC) compartment (Sharir et al., 2019). Use of different technical platforms [i.e., Smart-seq2 technology applied by Krivanek et al. (2020) which is characterized by higher sequencing depth than the 10× Genomics platform as used by Sharir et al. (2019) and Chiba et al. (2020)] may provide one explanation (Sharir et al., 2019; Chiba et al., 2020; Krivanek et al., 2020).

Additionally, through lineage tracing of *Notch1*^+^ SI cells in homeostatic conditions, Sharir et al. (2019) found that SI cells can contribute to both the SR and OEE lineages. When challenged by injury (i.e., disruption of the cycling progenitor population by 5-FU treatment or mechanical clipping of the incisor), *Notch1*^+^ SI cells rapidly (within 24 h) and drastically contributed to the proliferating progenitor cell pool as well as the ameloblast layer (Sharir et al., 2019). This finding suggests that injury triggers SI cells to enter the progenitor cell pool and contribute to tissue recovery. Interestingly, regeneration was also associated with increased expression of *Sfrp5*, previously described as a marker for the immediate progeny of *Sox2*^+^ DESCs (Juuri et al., 2012; Sharir et al., 2019). Together, the findings are in agreement with a highly heterogenous nature of dental epithelial stem/progenitor cells while challenging the classical tooth epithelial stem cell model in which a relatively small, slow-cycling population of DESCs in the SR and OEE gives rise to TA cells in the IEE that eventually differentiate into all dental epithelial lineages (Figure 5) (Sharir et al., 2019). In a revised model [also reviewed in Gan et al. (2020) and Fresia et al. (2021)], slow-cycling cells in the SR and OEE give rise to a large and heterogenous pool of constantly cycling progenitors located in the IEE, that in turn generate both ameloblast and non-ameloblast (including SR, OEE, and SI) lineages, thereby supporting the constant incisor growth and amelogenesis in homeostatic conditions. However, when demand is high as occurring during injury repair, existing SI cells contribute to the proliferating progenitor cell pool, as well as directly differentiate into both ameloblast and non-ameloblast epithelial cells. Similar concepts on stem/progenitor cell molecular and functional heterogeneity, and on mature cell de-differentiation and contribution during repair, have also been advanced in other tissues such as intestine and lungs (Tata et al., 2013; Murata et al., 2020).

### Wnt- and Shh-Responsive Dental Epithelial Stem Cells Subpopulations

Both Wnt- and Shh-responsive subpopulations have been identified in the heterogeneous DESCs (i.e., *Lgr5*- and *Gli1*/*Ptch1*-expressing cells). Hh signaling constitutes an essential driver for the continued generation of enamel-forming ameloblasts in the mouse incisor, but not for DESC self-renewal (Seidel et al., 2010). Intriguingly, the Shh signal was shown to originate from the differentiating DESC progeny, indicating a pro-differentiation feedback loop toward the DESCs (Seidel et al., 2010).

The role of Wnt/β-catenin signaling in DESC regulation has been more controversial and unclear (Liu and Millar, 2010; Suomalainen and Thesleff, 2010; Juuri et al., 2012). Currently, most evidence indicates inhibition of Wnt/β-catenin signaling in DESCs of the renewing incisor (Kuang-Hsien Hu et al., 2014). Firstly, several studies reported lack of canonical Wnt reporter activation in late-embryonic and postnatal laCL (Suomalainen and Thesleff, 2010; Juuri et al., 2012), as well as of *Axin2* expression in the E16.5 and E18.5 incisor DESC niche (Suomalainen and Thesleff, 2010). Secondly, multiple reports have identified expression of several Wnt antagonists in and around the laCL niche, such as *Sfrp5* (Juuri et al., 2012; Sharir et al., 2019; Chiba et al., 2020), *Sostdc1* (Sharir et al., 2019), *CD9* and LIM domain binding 1 (*Ldb1*) (Juuri et al., 2012), whereas only very few Wnt ligands could be detected in the dental epithelium of the laCL (Suomalainen and Thesleff, 2010). The Wnt antagonist *Sfrp5* displays a similar expression pattern as *Shh*, marking the early TA and pre-ameloblast progeny of the DESCs. However, in contrast to *Shh*, *Sfrp5* was also detected at the OEE/IEE junction of the CL tip, immediately surrounding *Sox2*^+^ DESCs (Juuri et al., 2012; Sharir et al., 2019; Chiba et al., 2020). Lineage tracing revealed that these *Sfrp5*^+^ cells are direct progeny of the *Sox2*^+^ DESCs (Juuri et al., 2012). Sc transcriptomics further supported this idea that the OEE/IEE junction contains direct descendants of tooth stem/progenitor cells (Sharir et al., 2019). Given that differentiating DESC progeny signals back toward their parent cells through Shh, it is not unlikely that secreted SFRP5 is also re-directed toward the DESCs to locally suppress Wnt/β-catenin activity. Furthermore, expression of non-degradable β-catenin in embryonic *Sox2*^+^ DESCs results in the formation of supernumerary tooth bud-like structures, or, when performed postnatally, in ectopic *de novo* tooth generation resembling odontoma (Xavier et al., 2015).

SOX2^+^ DESCs remain localized in the dental lamina [also referred to as the successional dental lamina (SDL)] throughout odontogenesis and are competent for successional tooth formation (Juuri et al., 2013). Tooth replacement occurs in the polyphyodont reptiles that continuously replace their dentition, or the diphyodont humans who form two generations of teeth (i.e., ‘milk’ and ‘permanent’ teeth). In monophyodont mice which only form one set of teeth, the dental lamina is involved in the sequential development of the three molars, after which it regresses to form a non-tooth forming rudimentary SDL (RSDL). The SOX2^+^ cells of the dental lamina give rise to (the epithelial compartment of) the second and third molars (Juuri et al., 2013; Yamakami et al., 2016). An analogous process appears to occur in other mammalian species with serial molar development, including humans and ferrets (Juuri et al., 2013). Interestingly, expression of *Sox2* and Wnt/β-catenin signaling (as shown by *Lef1* expression and nuclear β-catenin localization) are mutually exclusive in the SDL, being conserved throughout the animal kingdom, with activated Wnt/β-catenin in the tip of the SDL where *Sox2* expression is excluded (Handrigan and Richman, 2010; Gaete and Tucker, 2013; Juuri et al., 2013). Moreover, this juxtaposition of *Sox2*^+^/*Lef1*^–^ and *Sox2*^–^/*Lef1*^+^ domains is conserved through various stages of tooth development, including placode formation during which SOX2, together with PITX2, inhibits *Lef1* expression (Sun et al., 2016). In addition, it has been reported that *Lgr4* is co-expressed in the SOX2^+^ dental lamina cells of sequentially developing mouse molars, and is required to maintain SOX2 expression as well as stimulate Wnt/β-catenin activity and LEF1 expression in the dental lamina to safeguard the development of the sequential molars (Yamakami et al., 2016). Of note, *Lgr4* is also expressed throughout the DESC niche in the laCL and may be a DESC marker together with Lgr5 (Kawasaki et al., 2014). Thus, although not explicitly proven yet, the Lgr4-driven mechanism may be involved in the regulation of SDL and laCL DESCs. Taken together, Wnt/β-catenin activity at the SDL tip appears required to allow polarized elongation and proliferation of the SDL into the underlying dental mesenchyme, reminiscent of the potential, and similar, role for Wnt/β-catenin in the placode-to-bud and bud-to-cap transition during the earlier stages of odontogenesis (see above).

Similar to expression of constitutively activated β-catenin in SOX2^+^ DESCs in mice, overactivation of Wnt/β-catenin in snake dental organ cultures (through GSK3β inhibition) and in mouse SOX2^+^ RSDL (through expression of constitutively activated β-catenin) results in a supernumerary tooth phenotype (Gaete and Tucker, 2013; Xavier et al., 2015; Popa et al., 2019). Intriguingly, Shh plays a key role in restricting *Lef1* expression and Wnt/β-catenin to the distal SDL tip in snakes (Handrigan and Richman, 2010). It would be interesting to evaluate this Wnt-Shh interplay in mice, and to assess whether or not the conserved Wnt/Shh/Sostdc1/Lrp4 feedback is also ‘recycled’ here in this stage.

The mutual exclusivity of SOX2 and Wnt/β-catenin activity in the SDL, together with supportive evidence (including from other tissue systems) that SOX2 may exert an inhibitory effect on Wnt/β-catenin signaling, further underwrites the notion that Wnt/β-catenin inhibition is essential to keep the continuously renewing incisor DESCs in check (Okubo et al., 2006; Kelberman et al., 2008; Hashimoto et al., 2012; Sun et al., 2016). Intriguingly, *Sox2* has also been shown to lie downstream of Wnt/β-catenin signaling which may promote or inhibit *Sox2* expression depending on the context (Okubo et al., 2006; Hashimoto et al., 2012; Wang et al., 2013; Sun et al., 2016; Yamakami et al., 2016). The finding of a Wnt-responsive *Lgr5*^+^ subpopulation in the *Sox2*^+^ DESCs, as well as the (potential) feedback signals from the niche (e.g., Shh and SFRP5 from the DESC progeny), indicate the presence of a complex interactive system regulating stemness phenotype and function in the incisor epithelium. Of note, some studies have reported conflicting results, indicating activation (and not inhibition) of both canonical and non-canonical Wnt/β-catenin pathway in SOX2^+^ DESCs from mouse molars and incisors (Lee et al., 2016; Sanz-Navarro et al., 2018).

Together with the knowledge that, at previous developmental stages, both Wnt (hyper)activation and inhibition severely impair odontogenesis, and the fact that we currently lack data on the effect of Wnt inhibition on DESCs, it remains premature to draw firm conclusions on the role of Wnt/β-catenin signaling in DESCs.

### Wnt/β-Catenin and Shh Signaling in Dental Mesenchymal Stem Cells

It was long thought that the complete dental mesenchyme is solely derived from the cranial neural crest-derived ectomesenchyme (Miletich and Sharpe, 2004; Kaukua et al., 2014). However, it has recently been shown that both perivascular (i.e., pericytes) and glial cells (i.e., Schwann cells and Schwann cell precursors), also derived from the neural crest, can generate dental mesenchymal stem cells (DMSCs) which give rise to dental pulp and dentin-producing odontoblasts in a similar fashion as cranial neural crest (Feng et al., 2011; Kaukua et al., 2014; Yianni and Sharpe, 2019). The origin and diversity of DMSCs has been amply discussed in other reviews (Sharpe, 2016; Svandova et al., 2020). Here, we will focus on recent findings related to Wnt and Shh signaling in DMSCs.

As found in DESCs and its TA progeny, Gli1 is a marker of a quiescent population of DMSCs situated apically in between the laCL and liCL that continuously give rise to mesenchymal TA cells that contribute to the dental pulp and odontoblast lineages throughout the animal’s lifespan (Zhao et al., 2014; Krivanek et al., 2020). In incisors, *Gli1*^+^ DMSCs surround the incisor arteries and neurovascular bundle, and sensory neuron-derived Shh was shown to be a key regulator of DMSC homeostasis (Zhao et al., 2014). In concordance with the DESC niche, *Gli1*^+^ DMSCs and their TA cells represent juxtaposed, non-overlapping populations (Zhao et al., 2014; Jing et al., 2021). A recent study uncovered that the TA cells are characterized by *Axin2* expression and activated Wnt/β-catenin signaling, found crucial for TA cell proliferation and regulated epigenetically by the polycomb repressive complex 1 (PRC1) (An et al., 2018). Moreover, the TA cells play an essential role in the maintenance of the DMSC population *via* feedback signals. Of note, PRC1 has also been found to be a crucial regulator during molar root development, potentially via analogous mechanisms (Lapthanasupkul et al., 2012).

Several reciprocal interactions exist between the DMSCs and TA cell populations. Insulin-like growth factor 2 (IGF2) is secreted by *Gli1*^+^ DMSCs which activates Wnt/β-catenin signaling (e.g., increased *Axin2* expression) in TA cells *via* the IGF1 receptor (IGF1R), resulting in TA cell proliferation (Chen et al., 2020). Deletion of *Wls* in *Axin2*^+^ TA cells results in a reduction of both *Axin2*^+^ TA and *Gli1*^+^ DMSCs, indicating autocrine and paracrine feedback roles for TA cell-derived Wnt signals (such as TA-expressed *Wnt10a* or *Wnt5a*) (Jing et al., 2021). Deletion of *Wnt5a* from the *Axin2*^+^ TA cells, or of its receptor *Ror2* [receptor tyrosine kinase (RTK)-like orphan receptor 2] from *Gli1*^+^ DMSCs both resulted in a reduction of *Gli1*^+^ DMSCs and label-retaining cells, as well as disturbed dentinogenesis (Jing et al., 2021). Of note, a recent study further validated the requirement of ROR2 during dentinogenesis, as mesenchymal deletion of *Ror2* impaired odontoblast differentiation and led to shortened roots (Ma et al., 2021). Together, the findings support the existence of feedforward and -backward signaling between DMSCs and TA cells. Finally, *Gli1*^+^ DMSCs and *Axin2*+ progenitors are also found in other tooth compartments such as the PDL where they contribute to cementogenesis, or in developing molars, thus suggesting that identified mechanisms may be conserved in various DMSC populations throughout the tooth (Feng et al., 2017; Xie et al., 2019, 2021; Hosoya et al., 2020a).

## Conclusion and Relevance to Human Tooth Development and Other Fields

It is clear that Wnt/β-catenin and Shh signaling must be tightly balanced throughout the different stages of mouse tooth development. Both inactivation and hyperactivation of each one of these pathways lead to dental aberrations resulting in dysmorphic, missing or supernumerary teeth. Unfortunately, studies supporting these in-depth mouse-based findings in humans are sparse and are typically limited to gene and protein expression analysis, lacking mechanistic insight. However, also in humans, Wnt/β-catenin-activating and -inhibiting mutations, as well as mutations in components of the Shh pathway, have been linked to various tooth phenotypes (Table 1), thereby corroborating a key importance of these pathways during human tooth development. Mutations in these pathway components are mostly related to either familial, non-syndromic forms of tooth agenesis (including oligo- or hypodontia), or syndromic diseases with a dental phenotype, often representing a type of ectodermal dysplasia (i.e., syndromes characterized by defects in tissues of ectodermal origin such as teeth, hair, sweat glands, nails, eyes, mucous membranes and the central nervous system).

Our integrative review may also fertilize the field of dental tissue engineering and regeneration in which the discussed Wnt/β-catenin-Shh principles can be applied to achieve biomimetic structures (e.g., in combination with scaffold-based tissue engineering or organoid/assembloid approaches). However, as shown by the numerous studies discussed in the review, both pathways are very precisely regulated in time and space which will make clinical translation very challenging. Finally, given the highly similar development of tooth and other ectoderm-derived tissues (such as hair follicles, mammary glands, salivary glands, and palate), we expect that aspects and principles highlighted and discussed here will also be valuable for these other fields.

## Author Contributions

FH collected all the information, wrote the manuscript, and co-designed the figures. LH designed the figures and critically revised the manuscript. IL critically revised the manuscript. AB co-wrote and critically revised the manuscript. HV actively co-wrote, critically revised, and finalized the manuscript. All authors contributed to the article and approved the submitted version.

## Conflict of Interest

The authors declare that the research was conducted in the absence of any commercial or financial relationships that could be construed as a potential conflict of interest.

## Publisher’s Note

All claims expressed in this article are solely those of the authors and do not necessarily represent those of their affiliated organizations, or those of the publisher, the editors and the reviewers. Any product that may be evaluated in this article, or claim that may be made by its manufacturer, is not guaranteed or endorsed by the publisher.
